# Potential of Near Infrared Spectroscopy as a Rapid Method to Discriminate OTA and Non-OTA-Producing Mould Species in a Dry-Cured Ham Model System

**DOI:** 10.3390/toxins13090620

**Published:** 2021-09-03

**Authors:** Eva Cebrián, Félix Núñez, Mar Rodríguez, Silvia Grassi, Alberto González-Mohino

**Affiliations:** 1 Food Hygiene and Safety, Meat and Meat Products Research Institute (IProCar), Faculty of Veterinary Science, University of Extremadura, 10003 Cáceres, Spain; evcebrianc@unex.es (E.C.); fnunez@unex.es (F.N.); marrodri@unex.es (M.R.); 2 Department of Food, Environmental, and Nutritional Sciences (DeFENS), Università degli Studi di Milano, Via G. Celoria 2, 20133 Milan, Italy; 3 Food Technology, Meat and Meat Products Research Institute (IProCar), Faculty of Veterinary Science, University of Extremadura, 10003 Cáceres, Spain; albertogj@unex.es

**Keywords:** ochratoxin A (OTA), moulds, near-infrared spectroscopy (NIR), classification, portable device

## Abstract

The ripening process of dry-cured meat products is characterised by the development of fungi on the product’s surface. This population plays a beneficial role, but, uncontrolled moulds represent a health risk, since some of them may produce mycotoxins, such as ochratoxin A (OTA). The aim of the present work is to assess the potential of near-infrared spectroscopy (NIRS) for the detection of OTA-producing mould species on dry-cured ham-based agar. The collected spectra were used to develop Support Vector Machines–Discriminant Analysis (SVM-DA) models by a hierarchical approach. Firstly, an SVM-DA model was tested to discriminate OTA and non-OTA producers; then, two models were tested to discriminate species among the OTA producers and the non-OTA producers. OTA and non-OTA-producing moulds were discriminated with 85% sensitivity and 86% specificity in the prediction. Furthermore, the SVM-DA model could differentiate non-OTA-producing species with a 95% sensitivity and specificity. Promising results were obtained for the prediction of the four OTA-producing species tested, with a 69% and 90% sensitivity and specificity, respectively. The preliminary approach demonstrated the high potential of NIR spectroscopy, coupled with Chemometrics, to be used as a real-time automated routine monitorization of dry-cured ham surfaces.

## 1. Introduction

The environmental conditions during the ripening process of dry-cured meat products favour the development of fungi on the surface which become the predominant population [1,2]. Moulds play a beneficial role such as the reduction in rancidity [3], and the production of desirable volatile compounds due to their proteolytic and lipolytic activity, resulting in a strong impact on the development of the of the typical distinctive aroma and flavour of this type of product [4,5]. However, an uncontrolled mould development represents a high health risk, since some of them, usually isolated from dry-cured meat products, are able to produce mycotoxins.

Ochratoxin A (OTA) is the most concerning mycotoxin found in dry-cured meat products [6,7]. This mycotoxin is nephrotoxic, neurotoxic, genotoxic, teratogenic and has an immunosuppressive effect [8]. In addition, it has been rated by the International Agency for Research of Cancer (IARC) in the Group 2B as possibly carcinogenic to humans [9]. According to the EFSA Panel on Contaminants in the Food Chain, meat and meat products are among the main contributors of OTA exposure in the European Union (EU) [10]. This report may likely lead the EU to set a limit for OTA contamination in these foods. In this sense, Italy established a maximum value of 1 µg/kg for OTA in pork meat and derived products [11]. In this sense, several studies have shown that OTA is produced during ripening, and at the final product the amount of OTA is frequently higher than the guideline value [6,7,12]. Therefore, it is a real risk in dry-cured meat products that should be controlled at an early stage in their processing.

*Penicillium nordicum*, *Penicillium verrucosum* and *Aspergillus westerdijkiae* are commonly found in dry-cured meat products, being considered as the largest producers of OTA in these foods [7,13,14]. In this respect, the environmental conditions under which ripening occurs are within the range in which OTA is synthesised [15]. Thus, once the moulds start to develop, they can produce OTA until the end of processing, reaching very high levels of this mycotoxin [7]. Therefore, the early detection of OTA-producing moulds in dry-cured meat products is critical to prevent OTA reaching the food chain.

Increased efforts have been made to develop analytically simple, easy-to-use, relatively fast and easy portable methods suitable for rapid OTA screening [16]. However, from a food safety standpoint, and according to the HACCP approach, the detection of the OTA-producing moulds before OTA synthesis occurs is convenient, and it allows implementing corrective measures to prevent food contamination. Traditionally, fungal detection and identification has been determined by isolation and identification at the genus and species levels through macroscopic characteristics such as the colour, size and colony appearance as well as by microscopic characteristics [17]. However, it is a difficult and time-consuming process, and it also requires highly skilled personnel. Several DNA-based techniques, including PCR and LAMP methodologies, have been proposed as good alternatives to traditional identification, since they are quick, sensitive and specific, allowing for an accurate identification of the fungal ochratoxigenic species [18,19,20]. Nonetheless, these methods do not provide results in real-time, and require further sample processing in well-equipped specialised laboratories and trained staff. To overcome these limitations, a powerful, rapid, accurate, non-destructive and cost-effective method for a direct on-line detection of fungi on meat products should be developed.

In this sense, non-destructive spectroscopic techniques do not require sample preparation, having a high potential to be used as a real-time automated routine monitorization of dry-cured foods to prevent OTA contamination. In recent years, near-infrared (NIR) and mid-infrared (MIR) spectroscopy, as well as Raman spectroscopy, have been studied as promising tools for the detection of fungal contamination and the estimation of mycotoxin presence in foods [21]. Among the different commercially available NIR equipment, NIR portable devices are a good alternative to benchtop instruments, being equally reliable but cost-effective, faster and allowing in situ analyses. The advantages of these devices have been noted by other studies, remarking cost reduction [22] and a lower environmental impact [23] in comparison with benchtop ones. Therefore, thanks to this tool, it would be possible to monitor the development of toxigenic moulds and to establish the appropriate corrective measures in order to minimise the production of OTA at an early stage. 

Then, several methods based on NIR and MIR technologies, coupled with chemometric tools, have been used to discriminate healthy agricultural commodities from those contaminated with moulds of different genera such as *Aspergillus*, *Diplodia*, *Fusarium*, *Penicillium* or *Trichoderma* or with mycotoxins such as aflatoxins, OTA, fumonisin and deoxynivalenol [21,24]. In this sense, Fourier transform near-infrared spectroscopy (FT-NIR), combined with the Partial Least-Squares Discriminant Analysis (PLS-DA) and Principal Component-Linear Discriminant Analysis (PC-LDA), classification has proven to be useful for detecting and discriminating between different levels of OTA in cereals [25].

On the other hand, the hyphal wall of filamentous mould from Deuteromycetes, such as *Penicillium* and *Aspergillus* genera, is mainly composed of layers of polysaccharides such as β-glucans, chitin, galactomannans and glycosaminoglycans, along with glycoprotein and some lipids [26,27]. Then, the fungal cell composition and metabolism generate highly specific IR spectra which have been applied for the species identification of mould spore suspensions [28,29,30]. Accordingly, NIR and MIR spectroscopy have been proposed for the identification and characterization of filamentous fungi, including *Penicillium camemberti* grown on a cheese substrate [27], *Aspergillus* spp. in peanut [31] and grape-associated *Aspergillus* spp., *Botrytis cinerea* or *Penicillium expansum* [32,33].

Therefore, IR spectroscopy can be considered as a potentially useful method for the detection of OTA-producing moulds on the surface of dry-cured meat products to be used as part of the monitoring system in the industry for the prevention of this hazard. However, to the best of our knowledge, no work presents the use of portable devices for OTA-producing mould identification.

Thus, this work is a preliminary study that aims at exploring the efficacy of a portable NIR device in differentiating commonly isolated OTA- and non-OTA-producing moulds on a dry-cured meat-based substrate. The long-term objective is the development of a rapid test for the early detection of OTA-producing moulds in raw-cured meat products during processing.

## 2. Results and Discussion

In this work, the ability of NIR to discriminate OTA- and non-OTA-producing moulds was evaluated on a dry-cured ham-based agar (DHA) medium and at two temperatures (12 and 25 °C) for 32 days, simulating the usual conditions for the ripening of dry-cured meat products.

### 2.1. Mould Growth Characteristics

According to the visual assessment of the cultures, all mould strains used for this assay showed a satisfactory growth on the DHA medium at the two temperatures tested, showing no differences between OTA-producing and non-producing moulds. However, in general, a faster growth occurred at 25 °C than at 12 °C (Supplementary Figures S1 and S2).

### 2.2. MicroNIR Spectra and Principal Component Analysis

**Figure 1:**
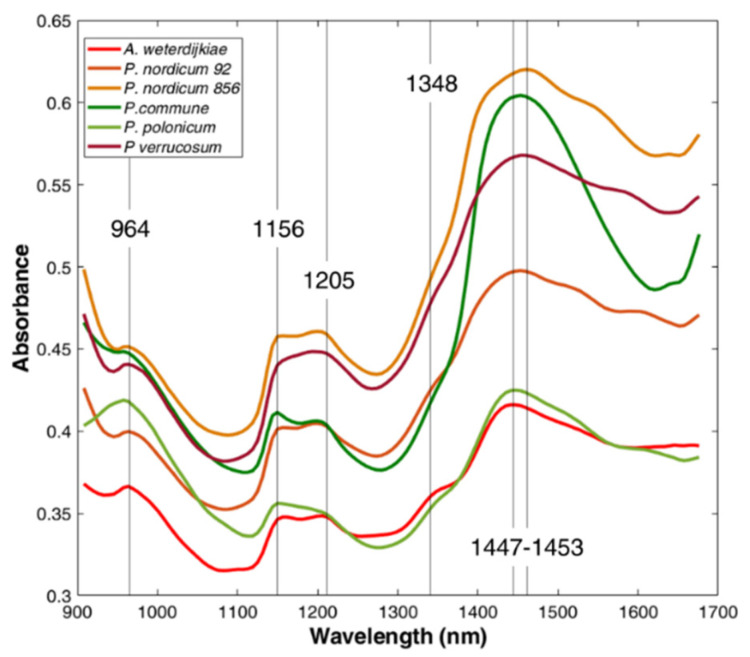
Averaged spectra for each species after 32 days of incubation at 25 °C. Non-OTA-producing species are greenish-coloured (*P. polonicum* and *P. commune*), whereas OTA-producing species (*A. westerdijkiae*, *P. nordicum* 92 and *P. nordicum* 856) are reddish-coloured.

Spectra were acquired by a portable device (MicroNIR OnSite spectrometer, VIAVI, Santa Rosa, CA, USA) in the spectral range 945–1500 nm with a 6.5 µs integration time and 100 scans. No differences were found among the NIR spectra of each mould at 12 and 25 °C. The acquired spectra were characterised by similar absorption bands and, to better discuss their characteristics, the average spectra of each mould after 32 days of incubation at 25 °C are reported in Figure 1. In detail, the average spectra showed specific bands at 964 nm, 1146 nm, 1205 nm and 1447–1453 nm, with a shoulder at 1348 nm. 

The 962 nm band was recognised as O–H alkyl alcohols with no hydrogen bonding (R-C-OH) [34]. The 1140 nm band was associated with the stretching of the C–O and C–C bonds to the second C–H harmonic spectrum and C–O–H, C–O–C angular deformations of carbohydrates of the fungi cell wall [35,36]. The region 1195–1215 nm was influenced by the absorption of C–H methyl functional groups as the second overtone of C–H [34]. This band has been associated with carbohydrates, in particular with the second overtone of CH stretching mode of carbonyl compounds [35,36]. At 1448nm it was reported as the N–H first overtone of aromatic amine [34]. Berardo et al. [35] assigned the signal between 1430 and 1470 nm to the first overtone of the OH stretching modes of glucose and NH in most amino acids.

Non-OTA-producing moulds were characterised by a slightly different absorption intensity. In particular, the band at 1156 nm was characterised by a higher absorbance in respect to the band at 1205 nm, whereas in the OTA-producing moulds, the two bands generally had the same absorbance, resulting in a more similar broad band than two distinct ones, especially for *P. verrucosum*. Moreover, *P. commune* was characterised by a different behaviour around 1450 nm; indeed, the band presented here a fast increase and decrease around its maximum.

**Figure 2:**
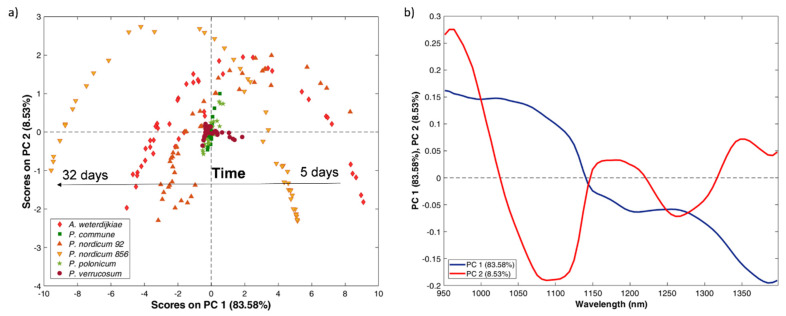
Principal Component Analysis developed from the spectra collected for the experiments conducted at 25 °C: (**a**) score plot of PC1 vs. PC2 where non-OTA-producing species are greenish-coloured (*P. polonicum* and *P. commune*), whereas OTA-producing species (*A. westerdijkiae*, *P. nordicum* 92 and *P. nordicum* 856) are reddish-coloured; (**b**) loading plot for PC1 and PC2.

Moreover, a PCA was performed to investigate the sample distribution according to the growing time. The spectra collected for each mould at a different sampling time for each tested temperature were merged in a dataset, reduced in the most informative spectral range (950–1400 nm) and pre-treated by smoothing and class mean centring. Figure 2 reports the score and loading plots obtained for the PCA built from the experiments performed at 25 °C. 

From the score plot (Figure 2a), it is possible to observe that OTA-producing moulds were characterised by a common behaviour and were distributed in all the quadrants of the plane defined by Principal Component 1 (PC1) and Principal Component 2 (PC2) with a time trend. Indeed, *A. westerdijkiae*, *P. nordicum* 92 and *P. nordicum* 856 describe a parabola: spectra collected at 5 days after the inoculum assumed positive PC1 scores; their scores decreased with the increment of time up to reaching negative values after 32 days. The parabolic behaviour derived from the simultaneous variation of PC2 according to the growing time; indeed, PC2 values for the sample collected at the beginning of the monitoring were negative; then, they increased to positive values but at the end of the monitoring they again assumed negative scores.

*P. verrucosum* behaviour appears different, with a small variation in the distribution of spectra collected according to time, and mainly along PC1. Even less variability can be observed for the non-OTA-producing species (*P. commune* and *P. polonicum*). All the spectra collected for the two moulds assumed PC1 scores close to zero; little variation can be observed along PC2 as their value increased with the progression of time. 

The samples distribution is well explained by the loading plot (Figure 2b), which describes the weight of each variable, the wavelength, in the space defined by PC1 and PC2. The loading of PC1 was meanly related to the scattering effect which affected the signal along the whole spectrum. This means that OTA-producing moulds were characterised by a higher growing rate in the tested conditions, resulting in a higher scatter effect on the collected spectra. On the other hand, PC2 loading showed an effect related to changes of the main absorption bands described above. In particular, the signals at 964 nm, 1146 nm, 1205 nm and 1348 nm were responsible for the displacement of samples to positive PC2 quadrants. Those differences are in accordance with the difference noticed in the spectral signal previously commented. Indeed, non-OTA-producing moulds were characterised by a higher absorbance at 1156 nm in respect to the band at 1205 nm, whereas in the OTA-producing moulds, the two bands generally had the same absorbance, resulting in a broad band.

The spectra characteristics together with the explorative analysis by PCA demonstrated that OTA and non-OTA moulds present different characteristics which could be the basis for a classification model development to discriminate these two classes.

### 2.3. Classification Models

At first, a Support Vector Machines–Discriminant Analysis (SVM-DA) classification model was developed to discriminate OTA-producing from non-OTA-producing species. Spectra were averaged on sample bases, pre-treated by smoothing and randomly divided into a calibration and a validation set, containing 124 and 77 samples, respectively. The calibration set was used to build the model and to internally validate the results by iterative cross-validation, whereas the validation set was used to externally validate the model; thus, miming its real-life application. The SVM-DA classification led to an optimal discrimination of the two classes in cross-validation (96% for sensitivity and specificity); however, the discrimination power reduced when performing the model validation in an external prediction (85% and 86% of sensitivity and specificity, respectively) (Table 1). 

In any case, the results were in line with the work reported by Fernández-Ibañez et al. [37], who investigated the utility of NIR spectroscopy for the rapid detection of aflatoxin B1 in both maize and barley. They were able to correctly classify 75% of their samples by using dispersive NIR.

More works report the development of classification models for a *Fusarium* damage evaluation on wheat kernels, even if those works were developed from spectra acquired by NIR-HSI systems. Indeed, Serranti et al. [38] used a general least square weighting algorithm (GLSW) as the pre-processing method and selected 12 effective wavelengths in three different ranges (1209–1230 nm, 1489–1510 nm and 1601–1622 nm) to construct a PLS-DA able to correctly classify Fusarium-damaged kernels (FDK) with a sensitivity and specificity range in cross-validation between 0.92 and 1.00. 

Similarly, Delwiche et al. [39] combined different wavelengths between 1000 and 1700 nm (1001.7, 1126.9, 1199.2, 1314.8, 1473.8 nm), obtaining an LDA classification accuracy of 82.5%. The same author developed a Vis/NIR-HSI protocol which was able to increase the classification ability obtaining a classification accuracy up to 95.0%. In the work by Williams et al. [40], the reliability of an NIR-HSI system in differentiating FDK of maize was also proved: they reached a classification accuracy between 94.0% and 97.7%. Furthermore, the recent study of Delwiche et al. [41] displayed percentages of a correct classification in a cross-validation higher than 92.0% when discriminating between sound and FDK by both LDA and PLS-DA. Among the proposed approaches, it is worth mentioning the industrial application developed by Pearson et al. [42]; they developed a discriminant analysis procedure able to correctly classify 97% of the kernels as contaminated (with >100 ppb of aflatoxin) and 100% of the kernels as uncontaminated (i.e., with no detectable aflatoxin). 

After the classification model developed to discriminate between non-OTA and OTA-producing moulds, the same spectra were used to develop other two classification models: one to discriminate among non-OTA-producing species and a second one to classify the different OTA-producing species considered. When moving to models developed to discriminate among species, good results were obtained in classifying non-OTA-producing species. Indeed, a 95% sensitivity and specificity were obtained in the prediction (Table 2) due to a misclassification of 2 out of 18 samples belonging to the *P. polonicum* class. 

Similarly, Da Conceição et al. [43] analysed by NIR-HSI (1000–2100 nm) two mycotoxicogenic *Fusarium* species. In detail, they considered twelve isolates of *Fusarium verticillioides* and three of *Fusarium graminearum* by growing them in 60 mm Petri dishes. Their model, developed on a pixel basis, achieved 100% accuracy, sensitivity and specificity. The better performance could be linked to the used system, characterised by a high spatial resolution (10 nm) thanks to images acquisition by a line-scan camera with a pixel size of 150 µm × 150 µm with a high-performance camera using a 50 mm lens and a field of view of 50 mm. 

The model developed for the discrimination among OTA-producing moulds did not perform as well as the previous one. The worst class assignment in prediction was observed for *P. nordicum* 92, where only 5 out of 10 samples were correctly predicted, whereas 2 samples belonging to this species were predicted as *P. nordicum* 856, 2 as *P. verrucosum* and 1 as *A. westerdijkiae*; thus, leading to a specificity of this class of 50%. However, the model gave a good global specificity in the prediction (90%) (Table 3). 

Therefore, NIR spectroscopy has great potential to discriminate the presence of OTA-producing mould species in meat substrates. Moreover, given its rapidity, simplicity, sensitivity and specificity, it could be used integrated in the HACCP in the food industry to the early detection of those moulds, allowing the implementation of preventive or corrective measures to prevent the OTA hazard in dry-cured meat products. In terms of costs, conventional species identification requires species isolation and a long-time of analysis with an average cost per each DNA-based analysis of around 40 euros. On the other hand, the NIR analysis only requires the equipment purchase (ranging from 10000 to 1000 euros according to the considered portable device) and the classification model development. Thus, the economic break-even-point is reached in mid-term, overcoming the economic impact of conventional analyses, together with an environmental advantage as already reported by Casson et al. [23]. In this sense, the obtained results are promising, but a larger data collection of moulds growing on meat products will strength the prediction reliability of the OTA-producing moulds model. Indeed, the model transfer to meat products should consider additional factors of variability, including irregular surfaces, a heterogenous meat composition, condensation presence, etc. 

## 3. Conclusions

In this study, we demonstrated that a portable device working in the near-infrared region is a useful tool for discriminating OTA-producing and non-OTA-producing mould species on a dry-cured meat-based substrate. Furthermore, SVM-DA models on the NIR spectra could be used for species screening purposes. The use of a portable NIR system could overcome the drawbacks related to DNA-based techniques for species discrimination, as it is easier and faster to execute, cost-effective against other tools and a non-destructive approach. In addition, since it is an easy-to-use technology, it could be carried out by the same staff who implement the HACCP system without the need for specific training.

The future testing of the developed approach directly on dry-cured ham surfaces could close the loop to provide the industry with a powerful tool for monitoring the safety of the dry-cured ham production chain, the results obtained in the present work promising to achieve this aim in final products. In any case, the model transfer would require the evaluation of additional factors of variability, such as irregular product surfaces, a heterogenous meat composition, native microbial population and condensation presence.

## 4. Materials and Methods

### 4.1. Moulds 

In this study, four ochratoxigenic moulds were used: *P. nordicum* CBS 323.92 from the Centraalbureau voor Schimmelcultures (The Netherlands), *P. nordicum* BFE 856 and *P. verrucosum* MRI 104 from the Federal Research Centre for Nutrition and Food (Karlsruhe, Germany) and *A. westerdijkiae* 6B/131, kindly supplied by Dr Paula Rodrigues from the Mountain Research Centre, Polytechnic Institute Bragança (Portugal). In the same way, two non-ochratoxigenic mould strains were used: *Penicillium commune* FHSCC 332 from the Food Hygiene and Safety Culture Collection at the University of Extremadura (Cáceres, Spain) and *Penicillium polonicum* CECT 20933 from the Spanish Type Culture Collection (Valencia, Spain). All of them were isolated from dry-cured meat products. 

### 4.2. Preparation of Moulds Inocula

Mould strains were maintained as stock cultures at −80 °C in PBS with 10% (*v/v*) glycerol as cryoprotectant. For this study, the inocula of each mould were prepared by growing on potato dextrose agar (PDA, Conda Pronadisa, Madrid, Spain) for 7 days at 25 °C. Conidia were harvested by rubbing the surface using saline phosphate buffer PBS; 0.32 g/L of NaH_2_PO_4_ (Scharlab S.L, Barcelona, Spain), 1.09 g/L of Na_2_HPO_4_ (Scharlab S.L.) and 0.9 of NaCl (Scharlab S.L.) with a glass rod. Spores were quantified by using a Thoma counting chamber BLAUBRAND^®^ (Brand, Germany) and adjusting to 10^7^ spores/mL to be used as inoculum.

### 4.3. Experimental Setting

This assay was carried out in DHA (30 g/L of lyophilised dry-cured ham, 20 g/L Bacto agar (Conda Pronadisa), 50 g/L of NaCl to simulate salt contents of dry-cured ham during ripening, and 1000 mL of distilled water). The water activity was 0.95. One hundred microlitres of each suspension of mould strain were inoculated by spreading it on the surface of the culture medium. The cultures were incubated at 12 and 25 °C for 32 days. At days 5, 13, 25 and 32 after inoculation, NIR measurements were performed at five different locations for each plate at the two study temperatures. Both the culture medium and the incubation temperatures were chosen to emulate the usual environmental conditions under which the moulds grow during the ripening of raw-cured meat products.

### 4.4. MicroNIR Spectra Acquisition 

**Figure 3:**
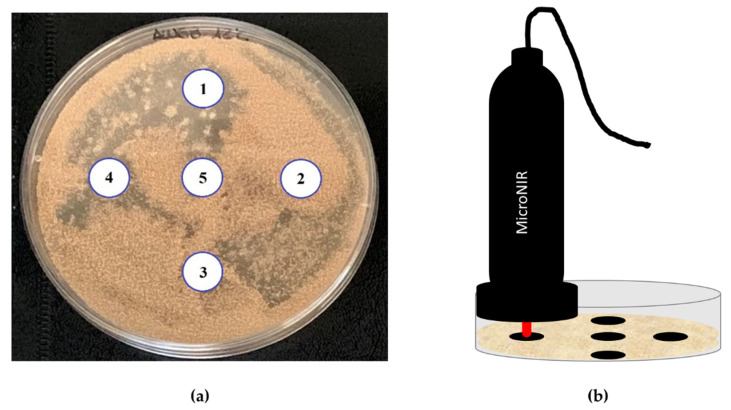
Sampling procedure: (**a**) example of the location of spectral acquisition points on a dry-cured ham agar dish inoculated with *Aspergillus westerdijkiae* 6B/131 and incubated for 32 days at 12 °C; (**b**) schematic acquisition procedure.

MicroNIR OnSite spectrometer (VIAVI, Santa Rosa, CA, USA) was used to analyse the plate with the DHA culture medium on the external surface of five locations, with a similar distribution to the picture reported in Figure 3a. In Figure 3b, a schematic representation of the sampling procedure is reported. The measurements were carried out with the contact of MicroNIR device and DHA medium; thus, the measurement distances were maintained in the different samples. The small size of the instrument (194 mm × 47 mm, weight <250 g) and the easy connectivity (by Bluetooth or USB) allowed a simple and fast (<0.25 sec per spectra) sampling procedure. Prior to analysis, the instrument was calibrated by the acquisition of the signal of a SPECTRALON^®^ as white standard. A disposable plastic was used between the device and the sample, which was replaced in each measurement. Spectra were acquired in the spectral range 945–1500 nm with a 6.5 µs integration time and 100 scans, with a spectral bandwidth lower than 1.25% of centre wavelength, typically 1% (e.g., at 1000 nm, the resolution is lower than 12.5 nm) and signal-to-noise ratio of 25,000. Spectra acquired were averaged on sample basis and merged in a unique dataset (201 samples × 125 wavelengths).

### 4.5. Data Analysis 

Spectral data were pre-treated by smoothing (Savitzky–Golay zero order polynomial, 5-points size) and mean centering. The dataset was divided, by the uniform sampling design procedure proposed by Kennard and Stone [44] into a calibration set used to build the model and a test set used to externally validate the model, containing 124 and 77 samples, respectively. The calibration set was also used to cross-validate the model in an iterative validation process for optimizing some model parameters, such as different number of factors. This was performed by iteratively removing 10% of samples from the calibration set and then building the classification model with the remaining samples, whereas the removed samples were used for model internal validation. Furthermore, the model was evaluated for blind prediction by external validation using the 77 samples removed from the collected data.

**Figure 4:**
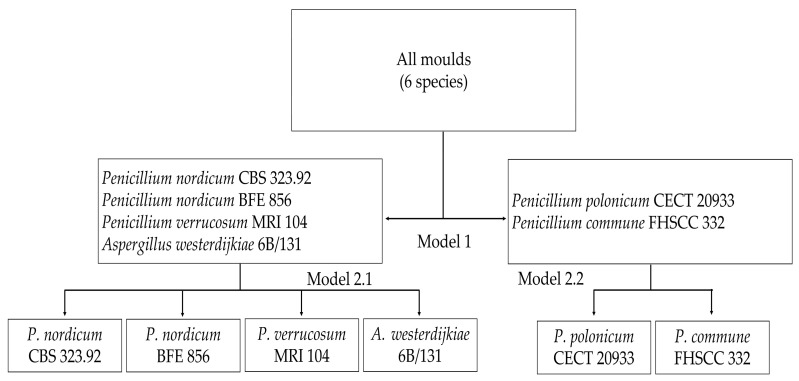
Scheme of hierarchic model development to classify OTA and non-OTA-producing moulds.

SVM-DA was applied to develop a series of classification models. In this context, a hierarchical approach was followed, considering at first a model to classify samples into OTA and non-OTA producers and then two models to discriminate species among the OTA producers and non-OTA producers (Figure 4).

SVM algorithm was applied for its ability to model nonlinear relations, choosing the C-SVC (C-support vector regression) algorithm which optimizes a model with an adjustable cost function (C), indicating how strongly misclassifications should be penalized. The parameters imposed to model 1 were: radial basis function as kernel type, upper tolerance on prediction errors (ɣ) equal to 3.16 and cost of prediction errors (C) equal to 100. Before classification modelling, a Principal Component Analysis (PCA) compression with 2 components was chosen to maximise the model stability and reduce the possibility to over-fit the data.

## Figures and Tables

**Table 1:** Figures of merit of SVM-DA model developed for OTA-producing moulds class prediction based on MicroNIR spectral data after smoothing.

		Sensitivity (%)		Specificity (%)	
		OTA	NON-OTA	OTA	NON-OTA
	*n* samples	63	61	63	61
Calibration	Class-based	98	100	100	98
	Average-based	99		99	
	*n* samples	63	61	63	61
Cross-validation	Class-based	95	97	0.97	0.95
	Average-based	96		96	
	*n* samples	40	37	40	37
Prediction	Class-based	76	95	95	76
	Average-based	85		86	

**Table 2:** Figures of merit of SVM-DA model developed for species classification among non-OTA-producing moulds based on MicroNIR spectral data after smoothing.

		Sensitivity (%)		Specificity (%)	
		*P. polonicum*	*P. commune*	*P. polonicum*	*P. commune*
	*n* samples	30	31	30	31
Calibration	Class-based	97	84	84	97
	Average-based	90		90	
	*n* samples	30	31	30	31
Cross-validation	Class-based	100	84	84	100
	Average-based	93		93	
	*n* samples	18	19	18	19
Prediction	Class-based	89	100	100	90
	Average-based	95		95	

**Table 3:** Figures of merit of SVM-DA model developed for species classification among OTA-producing moulds based on MicroNIR spectral data after smoothing.

		Sensitivity (%)				Specificity (%)			
		*P. nordicum 92*	*P. nordicum 856*	*P. verrucosum*	*A. weterdijkiae*	*P. nordicum 92*	*P. nordicum 856*	*P. verrucosum*	*A. weterdijkiae*
	*n* samples	25	23	20	20	25	23	20	20
Calibration	Class-based	60	91	70	95	92	91	94	94
	Average-based	78				93			
	*n* samples	25	23	20	20	25	23	20	20
Cross-validation	Class-based	60	91	70	90	92	91	91	96
	Average-based	77				92			
	*n* samples	10	13	18	17	10	13	18	17
Prediction	Class-based	50	85	56	82	92	87	95	85
	Average-based	69				90			

## Supplementary Materials

The following are available online at https://www.mdpi.com/article/10.3390/toxins13090620/s1, Figure S1: growth of different species of moulds in dry-cured ham-based agar for 5, 13, 25 and 32 days at 12 °C. *A. westerdijkiae* 6B/131 (AW 6B), *P. nordicum* BFE 856 (PN 856) and *P. nordicum* CBS 323.92 (PN 92), *P. polonicum* CECT 20933 (PP 20933) and *P. commune* FHSCC 332 (PC 332), Figure S2: growth of different species of moulds in dry-cured ham-based agar for 5, 13, 25 and 32 days at 25 °C. *A. westerdijkiae* 6B/131 (AW 6B), *P. nordicum* BFE 856 (PN 856) and *P. nordicum* CBS 323.92 (PN 92), *P. polonicum* CECT 20933 (PP 20933) and *P. commune* FHSCC 332 (PC 332).
