# Supplementary material for: Potential of Near Infrared Spectroscopy as a Rapid Method to Discriminate OTA and Non-OTA-Producing Mould Species in a Dry-Cured Ham Model System
Source: Toxins (Basel). 2021 Sep 3;13(9):620. doi: 10.3390/toxins13090620 (PMC8472122; doi:10.3390/toxins13090620)
Supplement: Supplementary file 1 [file toxins-13-00620-s001.zip › toxins-1289873-supplementary.pdf]

Supplementary Materials: Potential of Near Infrared Spectroscopy as a Rapid Method to Discriminate OTA and Non-OTA Producing Mould Species in a Dry-Cured Ham Model System

Eva Cebrián, Félix Núñez, Mar Rodríguez, Silvia Grassi and Alberto González-Mohino

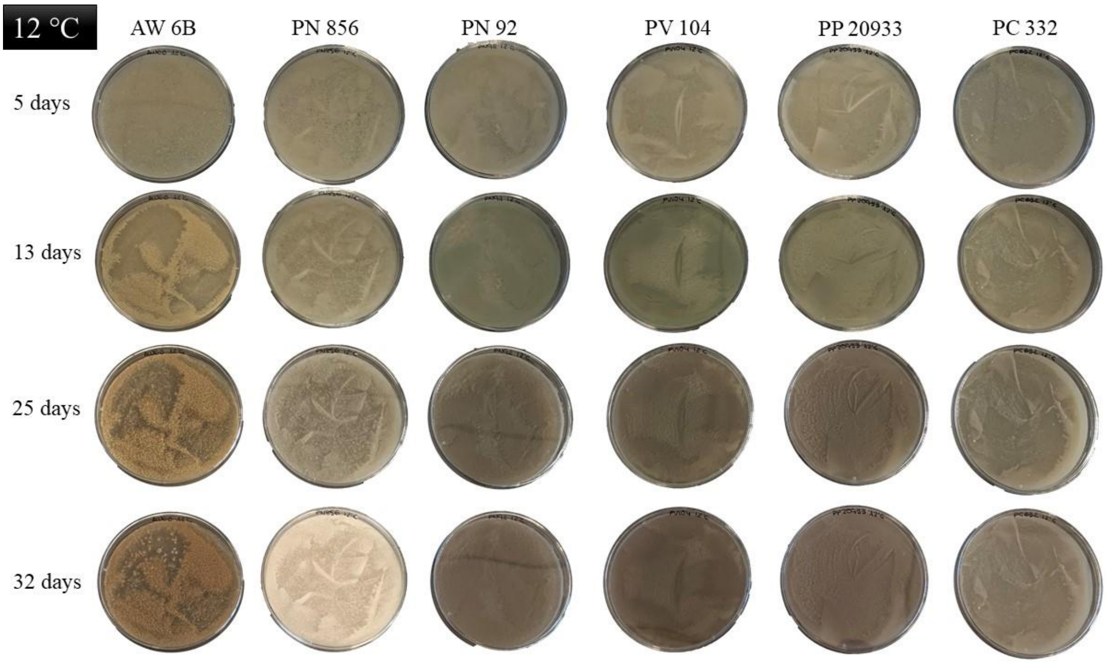

Figure S1. Growth of different species of moulds in dry-cured ham-based agar for 5, 13, 25 and 32 days at 12 °C. *A. westerdijkiae* 6B/131 (AW 6B), *P. nordicum* BFE 856 (PN 856) and *P. nordicum* CBS 323.92 (PN 92), *P. polonicum* CECT 20933 (PP 20933) and *P. commune* FHSCC 332 (PC 332).

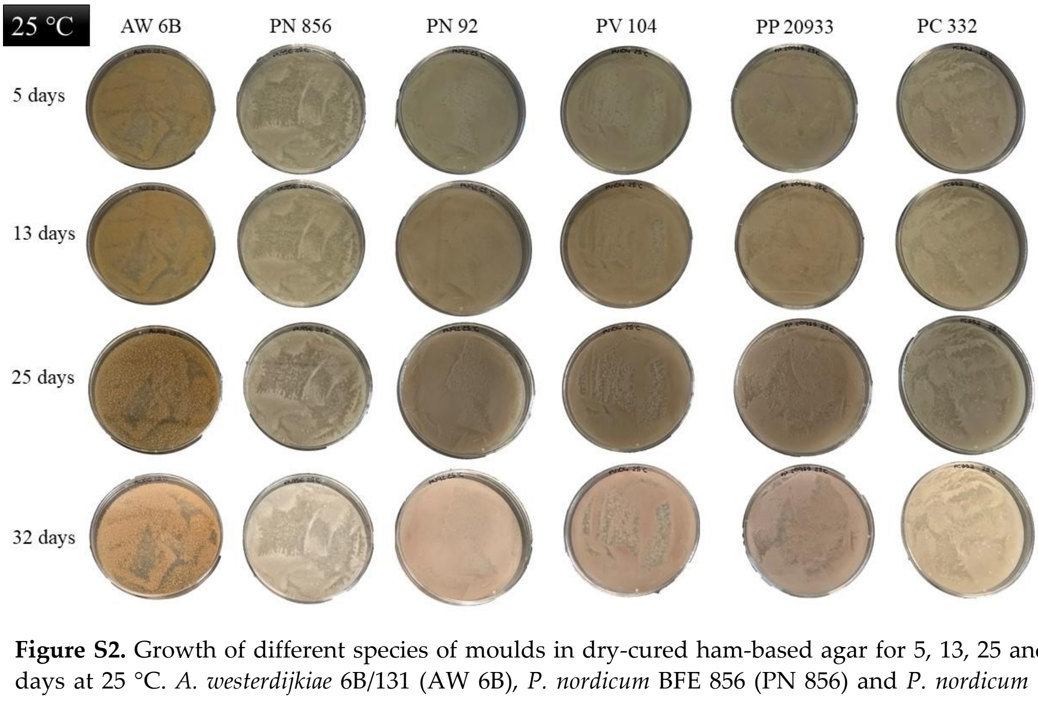

Figure S2. Growth of different species of moulds in dry-cured ham-based agar for 5, 13, 25 and 32 days at 25 °C. *A. westerdijkiae* 6B/131 (AW 6B), *P. nordicum* BFE 856 (PN 856) and *P. nordicum* CBS 323.92 (PN 92), *P. polonicum* CECT 20933 (PP 20933) and *P. commune* FHSCC 332 (PC 332).
